# Light extraction efficiency enhancement of flip-chip blue light-emitting diodes by anodic aluminum oxide

**DOI:** 10.3762/bjnano.9.152

**Published:** 2018-05-30

**Authors:** Yi-Ru Huang, Yao-Ching Chiu, Kuan-Chieh Huang, Shao-Ying Ting, Po-Jui Chiang, Chih-Ming Lai, Chun-Ping Jen, Snow H Tseng, Hsiang-Chen Wang

**Affiliations:** 1 Graduate Institute of Photonics and Optoelectronics, National Taiwan University, No. 1, Sec. 4, Roosevelt Rd., Taipei 10617, Taiwan; 2 Graduate Institute of Opto-Mechatronics, National Chung Cheng University, 168 University Rd., Min-Hsiung, Chia-Yi 62102, Taiwan; 3 R&D Center, Genesis Photonics Inc., No.5, Dali 3rd Rd., Shanhua Dist., Tainan City 74144, Taiwan; 4 Department of Electronic Engineering, National Kaohsiung University of Applied Sciences, No.415, Jiangong Rd., Sanmin Dist., Kaohsiung City 80778, Taiwan; 5 Department of Electronic Engineering, Ming Chuan University, Taoyuan 333, Taiwan,; 6 Department of Mechanical Engineering, National Chung Cheng University, 168 University Rd., Min-Hsiung, Chia-Yi 62102, Taiwan

**Keywords:** anodic aluminum oxide, critical angle of total reflection, efficiency enhancement, flip-chip blue light-emitting diode

## Abstract

We produced an anodic aluminum oxide (AAO) structure with periodic nanopores on the surface of flip-chip blue light-emitting diodes (FC-BLEDs). The nanopores had diameters ranging from 73 to 85 nm and were separated by distances ranging from approximately 10 to 15 nm. The light extraction efficiency enhancement of the FC-BLEDs subjected to different durations of the second pore-widening process was approximately 1.6–2.9%. The efficiency enhancement may be attributed to the following mechanism: periodic nanopores on the surface of FC-BLEDs reduce the critical angle of total reflection and effective energy transfer from a light emitter into a surface plasmon mode produced by AAO.

## Introduction

Light-emitting diodes (LEDs) are widely used in various fields, such as optical communication and automobile lighting [1–6]. The luminous efficiency of LEDs is defined as follows [7]. The luminous intensity of a 280 nm periodic structure in blue light and under a driving current of 700 mA is approximately 728.3 mW and is approximately 180 lm/W in white light [8–9]. Improving the luminous efficiency of LEDs is difficult. Ryu et al. prepared a scattering layer composed of fine inorganic particles. They then sprayed a planarization layer composed of acrylic acid resin or polyimide resin on the scattering layer and obtained a LED surface with high flatness and hardness; these properties enhanced the light extraction efficiency (LEE) of the LED [10]. Ding et al. fabricated a LED with enhanced LEE by depositing a patterned sapphire substrate (PSS) on a flip-chip LED with GaN as a base layer [11]. Shei et al. enhanced the LEE of LEDs by 27% by producing a microstructure on the surface of LEDs with Ga-doped zinc oxide (GZO)/GaN as the base layer and then reducing the total reflectivity by changing the shape, thickness, and density of the microstructure through dry etching [12]. Li et al. increased the LEE of an InGaN-monolayer quantum-well LED by 1.8–1.9 times relative to that of a traditional LED by producing a TiO_2_ microstructure array on p-GaN through dipping and rapid convective deposition and using noncrystalline TiO_2_ and anatase TiO_2_ with a diameter of 520 nm [13]. Huang et al. used Zn and Mg for ion implantation at the GZO thin layer and then adopted rapid thermal annealing to enhance the LEE of short-wavelength purple and ultraviolet LEDs by 1.4–2.5 times that of a traditional LED [14]. Ryu et al. adopted anodic aluminum oxide (AAO) to produce a photonic crystal structure at the n-GaN layer of LEDs, and transmission electron microscopy (TEM) images showed that screw dislocations in the LED structure were blocked, thus enhancing the general luminous efficiency by approximately 23% [15]. Cates et al. adopted laser etching to produce a repeated microstructure on the emitting surface of a yttrium aluminum perovskite scintillation crystal activated by cerium (formula YAlO_3_:Ce, abbreviated as YAP:Ce) and reduced the total reflection of light via surface roughening. The LEE of the LED was approximately two times that of traditional LEDs [16]. The use of a high-index-contrast photonic crystal [17–18] and colloidal-based microsphere arrays in conventional top-emitting LEDs [19], and microspheres and microlens arrays in thin-film flip-chip LEDs [20–22] and organic LEDs [23–25] has significantly improved the LEE. The use of microspheres and microlens arrays produced via the colloidal method has enabled the wafer-scale deposition of two-dimensional close-packed arrays with high density for GaN LEDs [19–22] and organic LEDs [23–25]. These technologies require multiple complicated processing methods or special materials and synthetic methods and are applied in LEDs with low luminous efficiency.

In the present study, AAO is used to produce a periodic nanostructures on high-luminance flip-chip blue LEDs (FC-BLEDs) to enhance the LEE [26–27]. This enhancement may occur through two mechanisms [28–34]. The nanostructure on the LED can decrease the total light reflectivity, thereby decreasing the critical angle of total reflectivity and enhancing LEE [12,16,35–36]. Whether the nanostructure, which possesses special metallic features, is influenced by the surface plasmon wave and enhances LEE has also been studied [37–40].

## Results and Discussion

### Scanning electron microscopy and atomic force microscopy measurements

**Figure 1:**
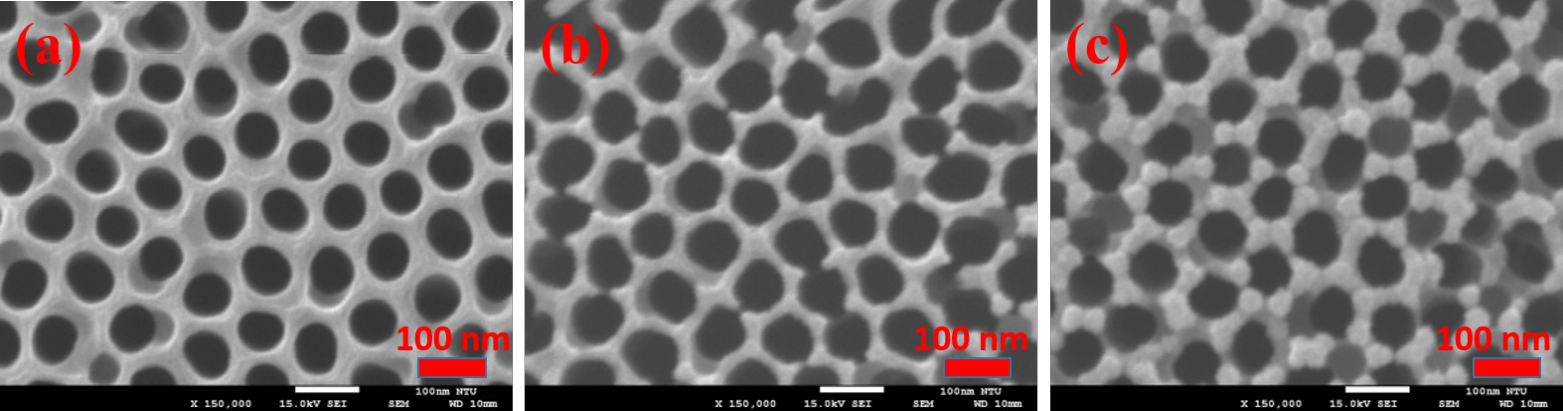
Scanning electron microscopy images of the anodic aluminum oxide (AAO) nanostructure on a sapphire substrate: sample (a) AAO60, (b) AAO70, and (c) AAO80.

The scanning electron microscopy (SEM) images of samples AAO60, AAO70, and AAO80 (whereby the numbers in the sample name indicate the duration of the pore-widening process, as described in the Experimental section) are shown in Figure 1a, Figure 1b, and Figure 1c, respectively. These figures show that the samples have a predominantly regular periodic nanostructure. The nanopore diameters of the three samples are approximately 73–75 nm, 77–79 nm, and 80–85 nm, and the distance between pores is approximately 15, 13, and 10 nm ± 2 nm. Figure 1a shows a relatively complete surface periodic structure; Figure 1b shows the presence of several fractures between pores, which are intensified; and Figure 1c shows a highly compromised structural integrity. The SEM results indicate that the pores gradually enlarge and that the distance between pores shortens as the duration of the second pore-widening process is extended. When the duration of the pore-widening process exceeds 70 min, the surface structure is damaged. Surface destruction, in turn, affects the integrity of internal pores. AFM measurements were then performed to determine AAO thickness, pore depth, and pore consistency.

**Figure 2:**
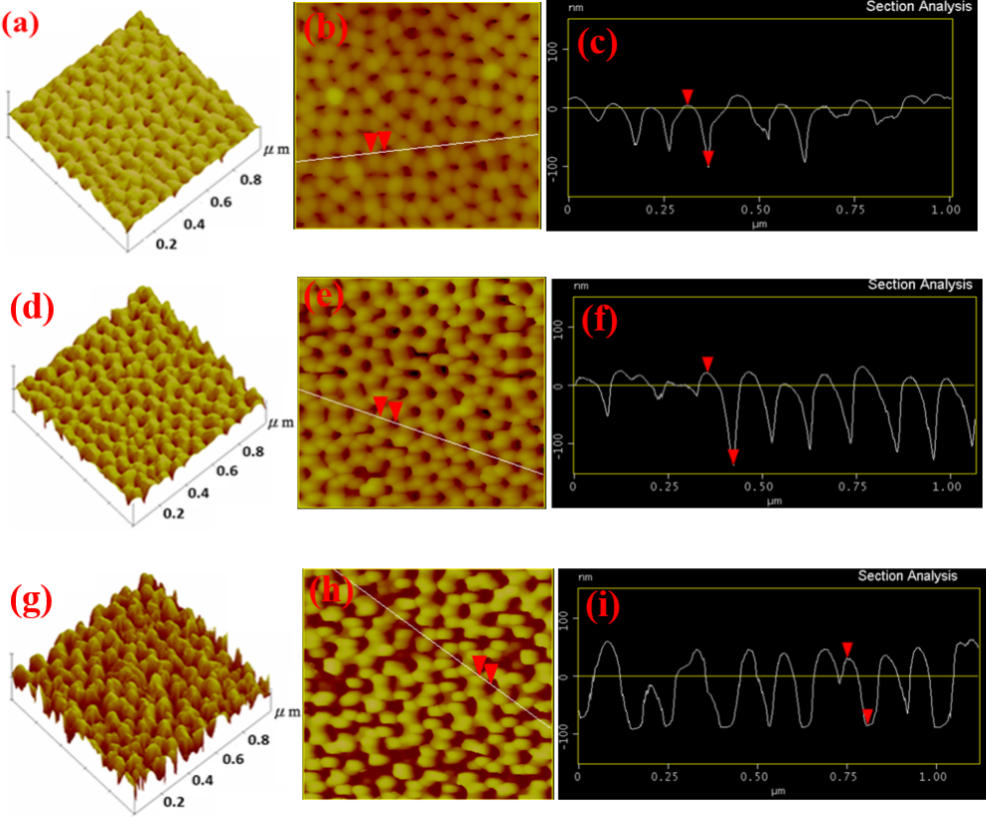
Atomic force microscopy measurements for samples (a–c) AAO60, (d–f) AAO70, and (g–i) AAO80.

The AFM results of the three samples are shown in Figure 2. The results show the surface roughness and surface height distribution of the three samples. Figure 2a, Figure 2d, and Figure 2g show the three-dimensional AFM images of AAO60, AAO70, and AAO80, respectively. Figure 2d shows the overall structure and structural integrity of the samples. The pores shown in Figure 2a have not widened completely, whereas those shown in Figure 2g have widened excessively. Excessive widening destroys the surface structure. The AFM images of the three samples are shown in Figure 2b,e,h and were used to examine surface integrity, whereby pores are not evident in Figure 2b. Figure 2h shows pores that have been etched excessively. Excessive etching destroys the surface structure. Figure 2e shows pores that have been completely etched. These results are consistent with the SEM measurements. We measured the line-scan profile of the AFM images to determine the correlation between the depth and width of the internal structure, as well as structural distribution. The results are shown in Figure 2c,f,i. AAO60 presents a surface depth difference of approximately 50 ± 2 nm, pore diameter of approximately 75 ± 3 nm, and the depth-to-width ratio of approximately 2:3 (see Figure 2c). The surface structure of AAO60 is uneven and complete. AAO70 presents the surface depth difference of approximately 100 ± 3 nm, pore diameter of approximately 78 ± 3 nm, and the depth-to-width ratio of approximately 4:3 (see Figure 2f). Moreover, an indistinct structure has been generated between 0–0.4 µm of the *x*-axis (assuming that the sampling position is between the pores). Beyond 0.4 µm, the structural periodicity is extremely homogeneous and pore depth is consistent. AAO080 presents a surface depth difference of approximately 100 ± 3 nm, pore diameter of approximately 85 ± 2 nm, and a depth-to-width ratio of approximately 20:17. The depth distribution of pores is extremely homogenous (see Figure 2i). However, two neighboring pores 0.25 µm from the measurement position are connected. Thus, destructive etching may occur on the surface. These results showed that as the duration of the pore-widening process is extended, the diameter of AAO pores on the LED increases from 75 nm to 85 nm, the surface depth increases from 50 nm to 100 nm, and the depth-to-width ratio approaches 1:1. These effects result in surface destruction and pore connection. The above results indicate that AAO60 does not undergo inadequate etching. By contrast, AAO80 undergoes overetching, which causes surface destruction. Consistent with the SEM measurements, AAO70 exhibits the optimal pore periodicity.

### Transmission electron microscopy measurements

**Figure 3:**
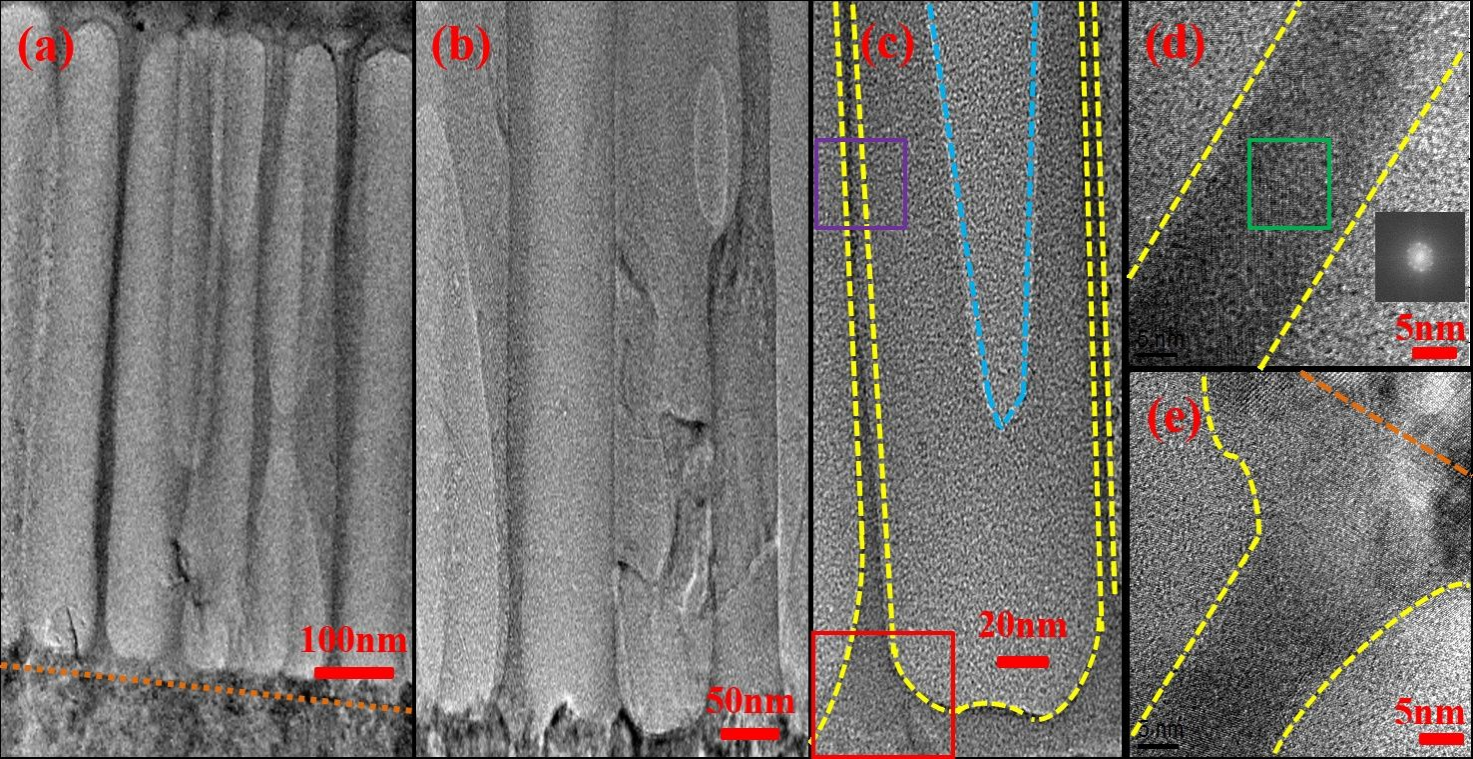
Transmission electron microscopy measurements for sample AAO60 at different magnifications: (a) complete pores exist at the sapphire substrate; (b) a magnified view of (a); (c) a magnified view of the single pore shown in (b); (d) a magnified view of the region defined by the purple rectangle in (c); and (e) a magnified view of the red rectangle region shown in (c).

**Figure 4:**
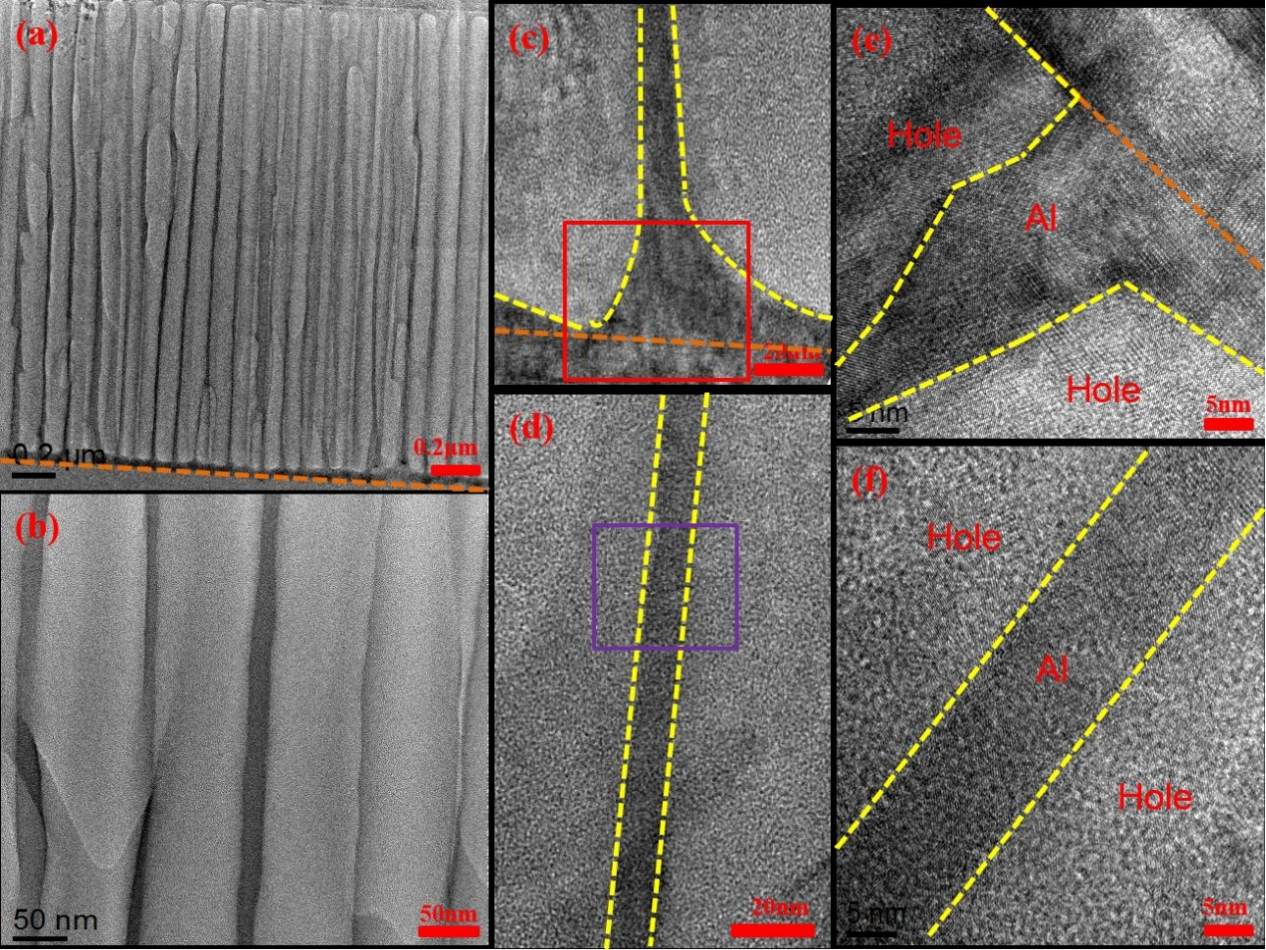
Transmission electron microscopy measurements for sample AAO70 obtained at different magnifications: (a) complete pores exist at the sapphire substrate; (b) a magnified view of (a); (c, d) magnified views of the areas between the pores shown in (a); (e) magnified view of the red area in (c); and (f) magnified view of the purple area shown in (d).

**Figure 5:**
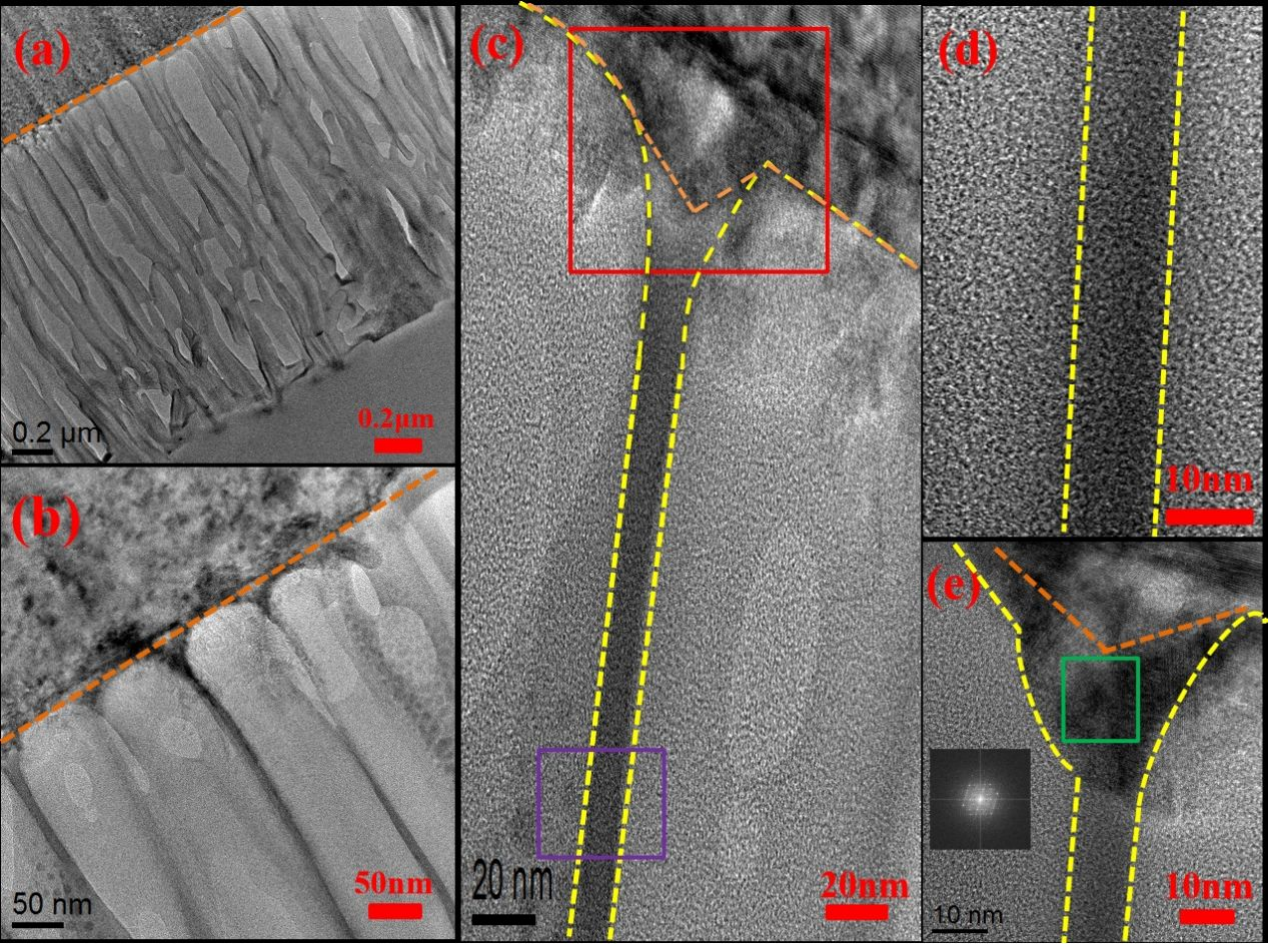
Transmission electron microscopy measurements for AAO80 obtained at different magnifications: (a) complete pores exist at the sapphire substrate; (b) magnified view of (a); (c) the structure between pores; (d) a magnified view of the purple area shown in (c); (e) a magnified view of the red area shown in (c).

To understand the internal structure and elemental distribution of the nanopores, the TEM images of the three samples were acquired. The images are shown in Figure 3, Figure 4, and Figure 5. In these figures, the measurement scale is accurate in the *x*-direction, whereas in the *y*-direction, it is magnified to help identify the integrity of the pore structure in this direction. In Figure 3e, the orange dotted line represents the boundary between pores and the sapphire substrate of AAO60. The area bounded by the yellow dotted line is Al, the area bounded by the blue dotted line is a pore, and the area between these lines is Al_2_O_3_. In Figure 3a, most pores are etched completely and are connected to the sapphire substrate. Figure 3b provides a magnified view of Figure 3a and shows the AAO pore structure in detail. In this figure, some pores are etched completely, while others are not. Figure 3c–e shows the internal elemental distribution in individual pores. Figure 3c provides a magnified view of the single pore shown in Figure 3b. The detailed boundary of the pore is defined by dotted lines. Figure 3d shows a magnified view of the region defined by the purple rectangle in Figure 3c. The inset shows the diffraction pattern based on the fast Fourier transform of the green section in Figure 3d. The figure shows that the lattice structure is as regular as the hexagon lattice structure of Al. Figure 3e shows a magnified view of the red rectangle region shown in Figure 3c. This figure indicates that some pores have not been completely etched.

Figure 4a shows that most pores of AAO70 have been etched completely and are connected to the sapphire substrate. Moreover, the overall integrity of the pores is higher than that shown in Figure 3a. Figure 4b shows the TEM image of a higher rate. The pore integrity of AAO70 is superior to that of AAO60. Figure 4c and Figure 4d are magnified views of the areas between the pores shown in Figure 4a. Figure 4e is a magnified view of the red area in Figure 4c and shows that the pores are connected to the sapphire substrate. The three areas included an etched hole, Al, and the sapphire substrate. Figure 4f is a magnified view of the purple area shown in Figure 4d. The Al lattice structures are bounded by the yellow dotted line, with pores existing beyond the dotted line. Figure 5a shows that the pores of AAO80 have been etched completely and are connected to the sapphire substrate. However, most pores have been etched and destroyed internally, and their structures are not complete. Figure 5b shows the AAO pore structure in detail. Figure 5c–e shows the internal elemental distribution of individual pores. Figure 5c shows the structure between pores, and Figure 5d is a magnified view of the purple area shown in Figure 5c. In Figure 5d, the area bounded by the yellow dotted line is Al. Figure 5e provides a magnified view of the red area shown in Figure 5c. The figure shows that the pores are connected with the substrate. We selected the green area in Figure 5e for lattice structure analysis. The results are shown as the inset figure in the image. The area bounded by the yellow dotted line and the orange dotted line at the bottom is a regular hexagonal lattice structure. TEM analysis results show that AAO70 has been completely but not excessively etched. By contrast, AAO60 has been incompletely etched, and AAO80 exhibits structural destruction caused by excessive etching. Thus, different pore-widening durations cause the pore integrity to vary among the samples. Excessive pore-widening duration can cause pore destruction, which influences LED luminous efficiency.

### Optical measurements

**Figure 6:**
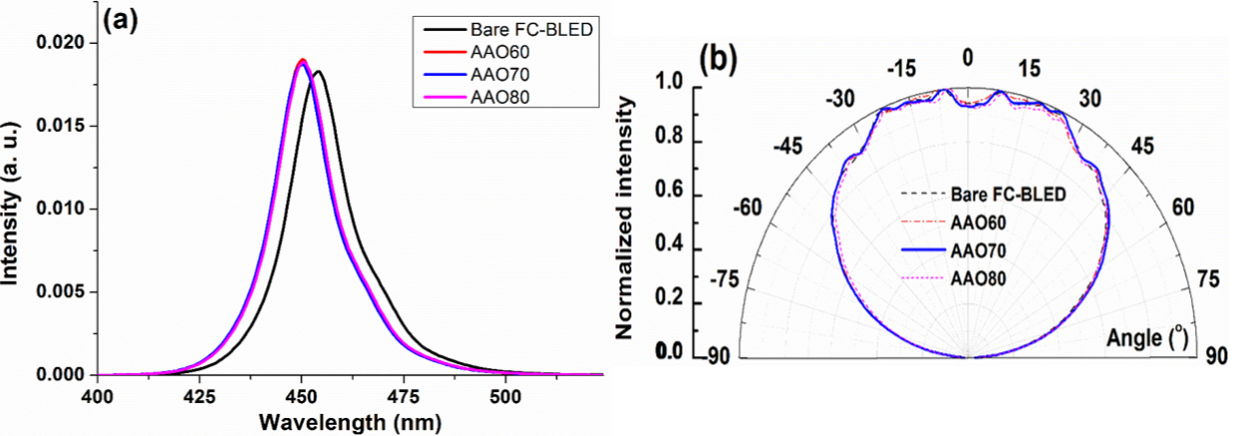
(a) Measured luminous wavelength versus energy and (b) luminous angle for samples AAO60, AAO70, AAO80, and bare FC-BLED.

**Figure 7:**
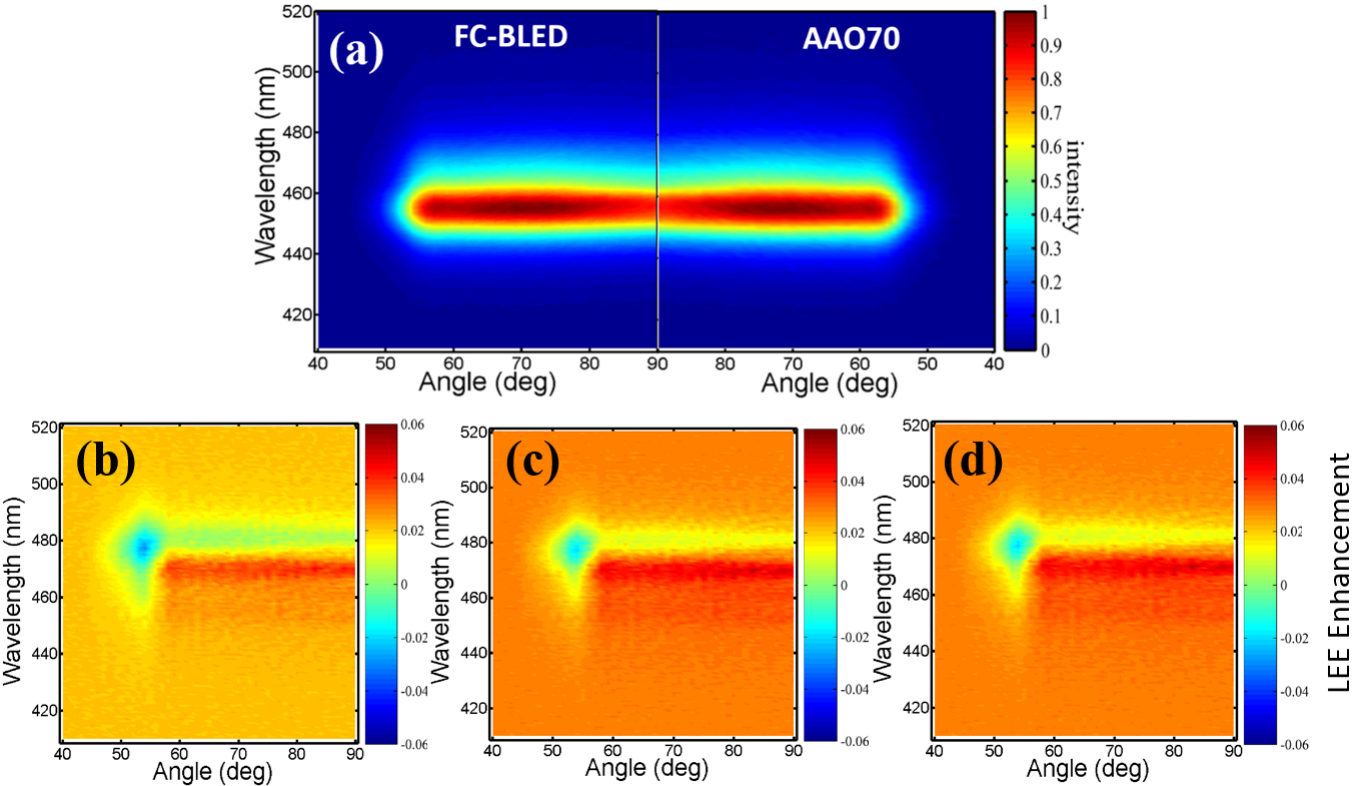
(a) Angle-resolved electroluminescence (AREL) spectra of samples FC-BLED and AAO70. Angular resolution of LEE enhancement of (b) AAO60, (c) AAO70, and (d) AAO80.

Figure 6a shows the measured FC-BLED luminous wavelength and radiation angle of a structure-less crystal-covering type of LED, AAO60, AAO70, and AAO80. The LED with an AAO structure has higher luminous intensity than the LED without an AAO structure. The wavelength exhibits a blue shift of approximately 5 nm. The following two mechanisms can explain the blue shift: First, InGaN multiquantum wells (MQWs) grow because of patterned sapphire substrate (PSS) technology and the luminous blue shift is generated by compressive strain release [26]. However, this study does not consider the contribution of this phenomenon. Second, the AAO process introduces the surface-plasmon-induced blue shift of LED emission between Al and the sapphire substrate [37,41–45]. Figure 6b shows that the luminous angle for the test block in AAO70 is the largest. The inadequate pore-widening duration of AAO60 results in inadequate pore etching, thereby influencing the surface luminous intensity and radiation angle of AAO60. The excessive pore-widening duration used to fabricate AAO80 causes the surface destruction of pores and thus resulted in the poor luminous intensity and small radiation angle of AAO80. Therefore, the optimal radiation angle and luminous intensity can be obtained with the pore-widening duration of 70 min (i.e., AAO70). To better understand the mechanisms underlying the blue shift of emission energy, the angle-resolved electroluminescence (AREL) spectra of the samples were collected and are shown in Figure 7a. The angular plots of all samples show broad protrusions between −50 to 50°. The electroluminescence (EL) intensity of AAO70 is approximately 2.9% higher than that of the bare FC-BLED. The AREL spectra of the samples are not significantly different. AREL results were calculated as follows:

[1]
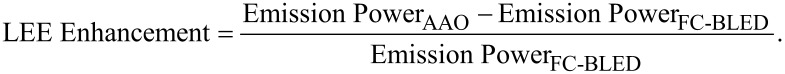


**Figure 8:**
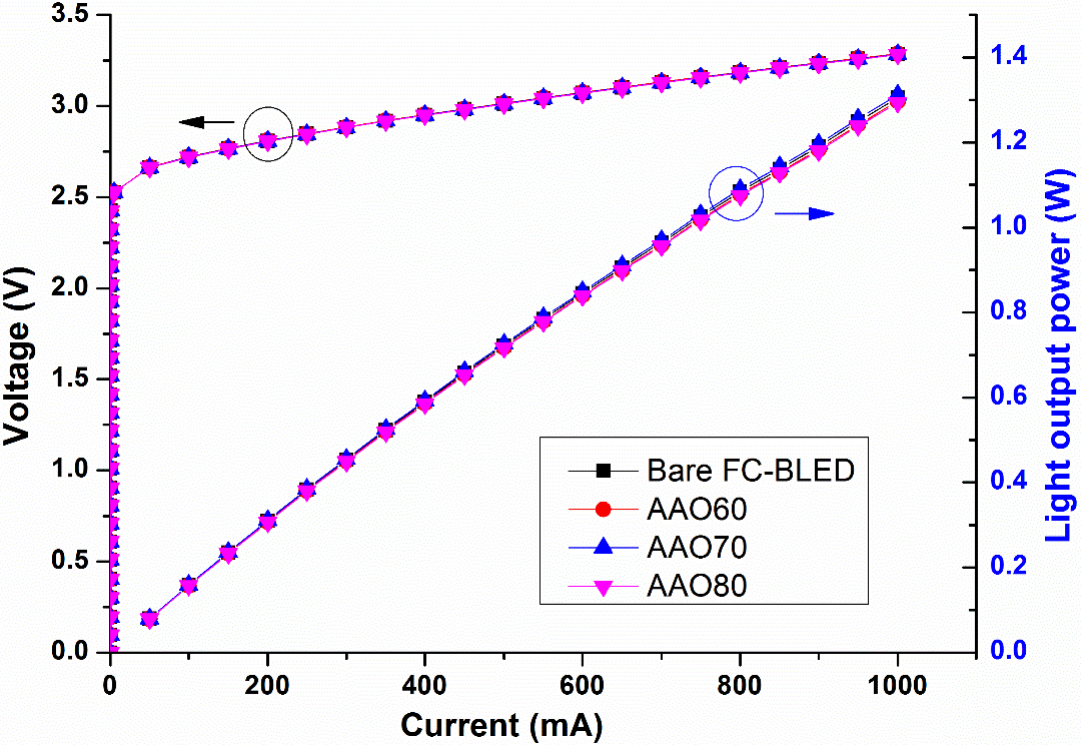
Typical light output power–current–voltage (*L*–*I*–*V*) curves of the samples in this study.

Figure 7b–d shows the angular resolution of the LEE enhancement of AAO60, AAO70, and AAO80. In Figure 7b–d, the LEE enhancement is negative at the angles of 50–60° and energies of 455–480 nm given the blue shift of emission energy in the AAO-structured samples. Positive LEE enhancement values are obtained at angles of 55–90° and energies of 425–455 nm. Figure 6a shows the same tendencies for these values. Figure 7b shows that, consistent with the other optical measurements, AAO70 has the largest LEE enhancement values. The measurement results of AAO60, AAO70, and AAO80 are shown in Table 1. The number of test blocks is 30. Extending the second pore-widening duration improves the efficiency enhancement. However, the luminous efficiency of AAO80 is inferior to that of AAO70 because the internal structure of the former has been destroyed. The results in Table 1 show that among all samples, AAO70 has the highest luminous rate, shortest luminous wavelength, and largest radiation angle. These improvements resulted from subjecting the LED surface to AAO. The light output power of the LED devices is measured using an integrating sphere system. Figure 8 shows the typical light output power–current–voltage (*L*–*I*–*V*) characteristics of the LEDs with and without AAO structures. The AAO structures enhance the LEE of LEDs but do not change the *L*–*I*–*V* properties.

**Table 1:** Light output power (LOP), emission peak, radiation angle, and light extraction efficiency (LEE) enhancement of bare FC-BLEDs, samples AAO60, AAO70, and AAO80.

Device	LOP (mW) at 350 mA	Emission peak (nm)	Radiation angle (°)	LEE enhancement (%)

Bare FC-BLEDs	407.3 ± 0.8	453.8 ± 0.1	133.8 ± 0.7	–
Sample AAO60	416.2 ± 5.0	451.0 ± 1.0	134.4 ± 0.3	2.1
Sample AAO70	419.3 ± 0.7	450.4 ± 0.5	138.3 ± 1.0	2.9
Sample AAO80	414.0 ± 1.0	450.5 ± 0.5	137.6 ± 0.5	1.6

### Rigorous coupled-wave analysis

**Figure 9:**
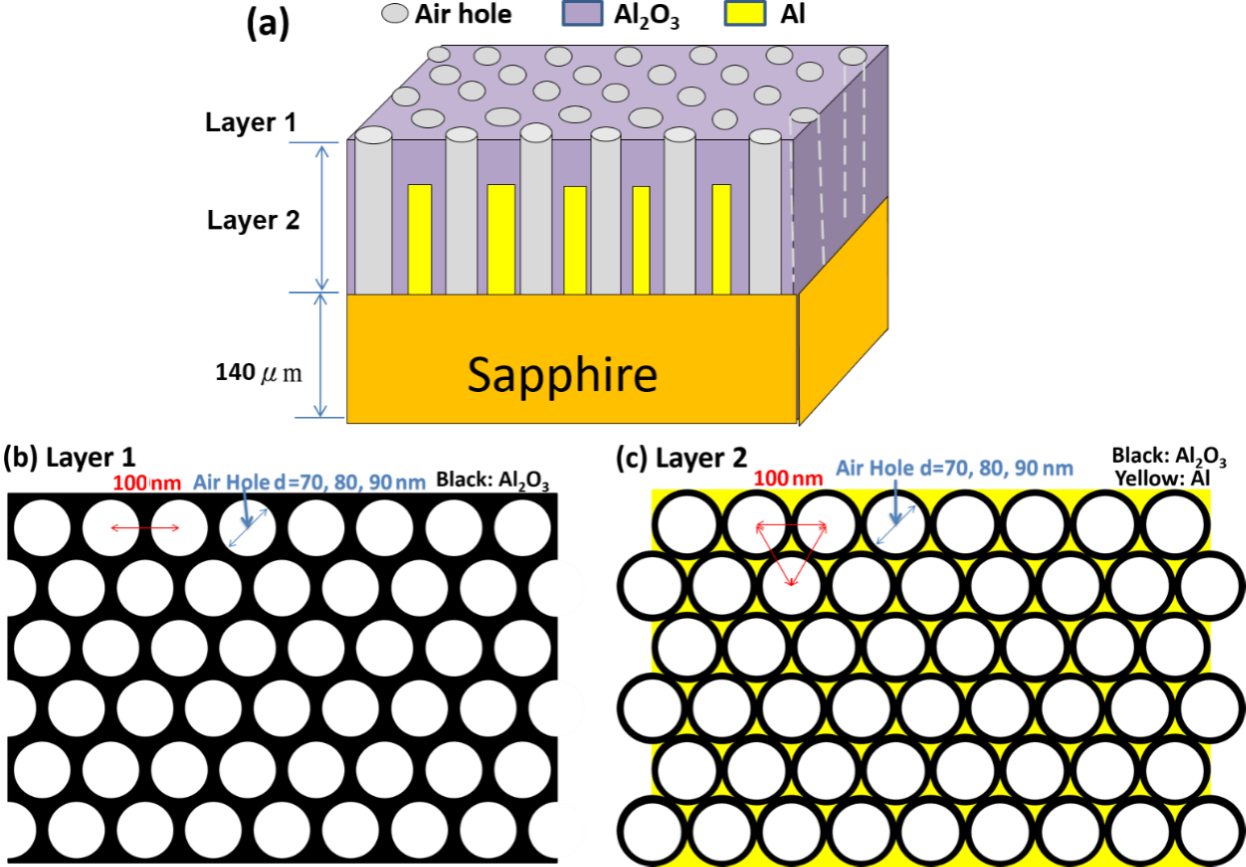
Sample structure used in rigorous coupled-wave analysis (RCWA) calculations: (a) AAO sample structure, (b) layer 1 structure and (c) layer 2 structure.

**Figure 10:**
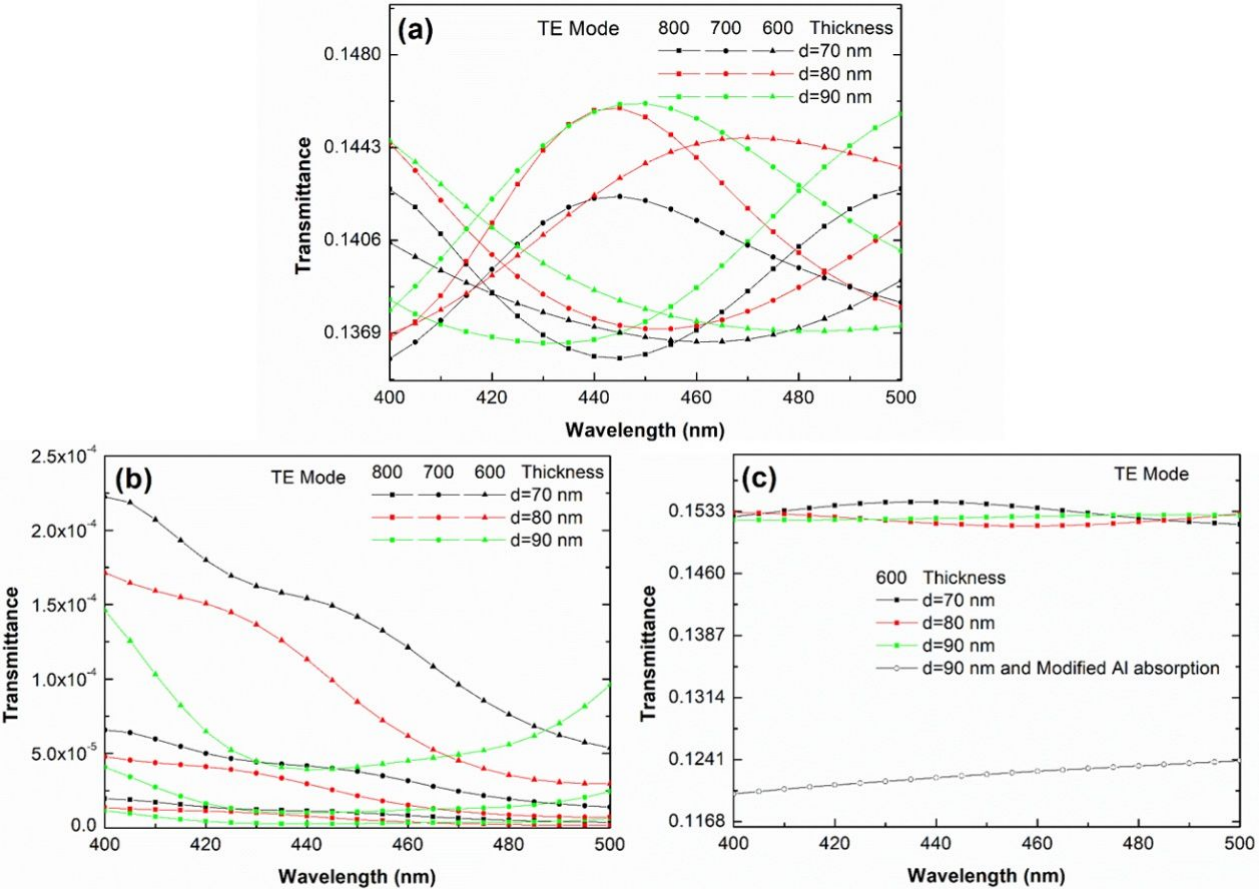
Rigorous coupled-wave analysis (RCWA) results obtained with different Al_2_O_3_ thickness and air-hole diameters (a) without Al (b) with Al and (c) with modified Al absorption.

**Figure 11:**
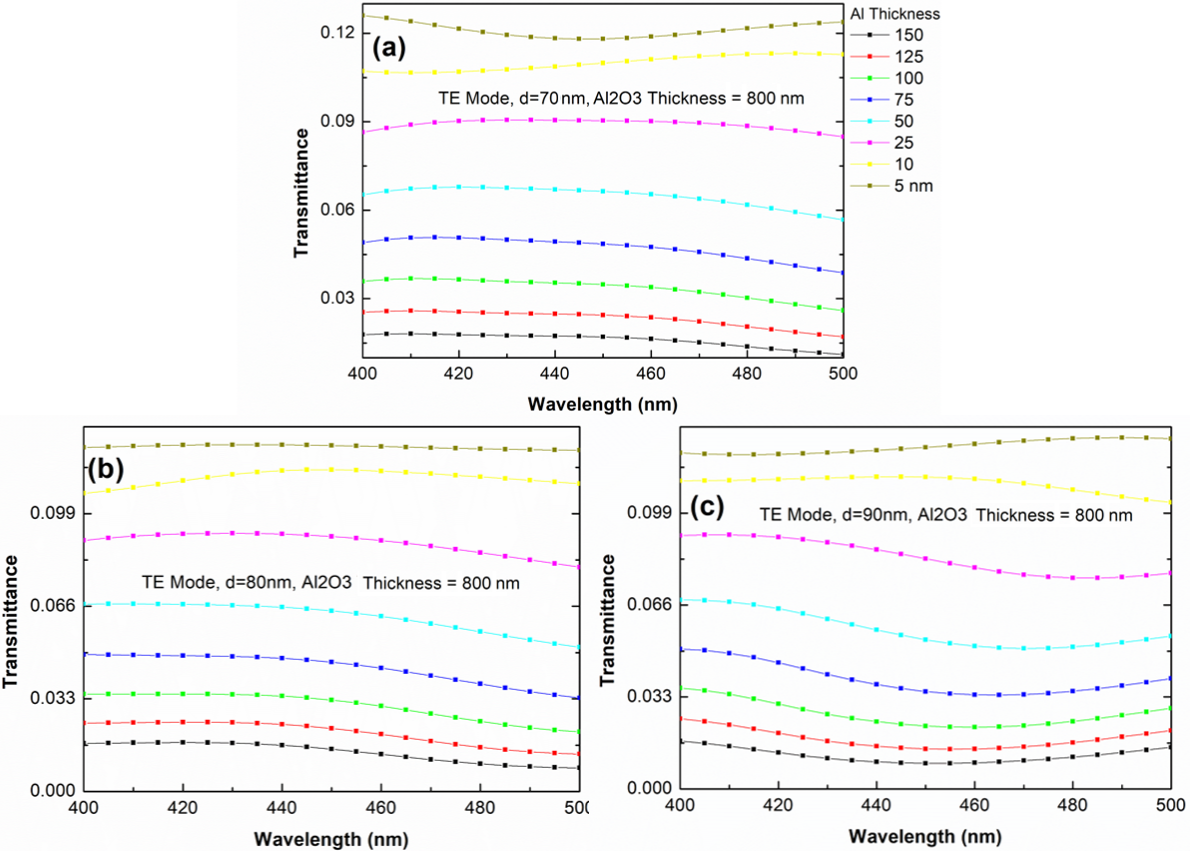
Rigorous coupled-wave analysis (RCWA) results with different Al thickness values and fixed Al_2_O_3_ thickness of 800 nm for samples (a) AAO60 (b) AAO70 and (c) AAO80.

We used the rigorous coupled-wave analysis (RCWA) to verify our hypothesis and to clearly explain the reduction in total reflectivity and the existence of the surface plasmon wave in the samples. RCWA is a semianalytical method in computational electromagnetics that is typically applied to solve scattering from periodic dielectric structures [46]. Figure 9a shows the sample structure, which is constructed on the basis of SEM results, used in this calculation. Figure 9b and Figure 9c show layers 1 and 2 as the calculation layers, respectively. Air hole diameters are set as the control parameters and are equal to the size of AAO60, AAO70, and AAO80. In Figure 9c, the diameter of Al_2_O_3_ is 100 nm, which is consistent with the hole radius. If the hole radius decreases, then the yellow area (Al) will expand. Figure 10a shows the calculation results obtained with different Al_2_O_3_ thicknesses and diameter values of air holes in the absence of Al. All the three samples have the same transmittance of 440–450 nm. However, AAO80 has wider transmittance windows than other samples. Figure 10b shows that transmittance drastically decreases when Al is present in the sample structure. The thickness of Al in the form of Al_2_O_3_ is less than 10 nm. Figure 10c shows the modified absorption result for Al after the refractive index of the imaginary part (absorption) has been multiplied by 0.01, that is, the artificial absorption of Al has decreased by 100 times. In this condition, the transmittance of the modified Al absorption is still lower than without Al calculations. This type of calculation result indicates reasonable physics. Extremely low transmittance exists when the thickness of Al is approximately 790–590 nm. We redesigned the sample structure with different Al thicknesses in reference to the TEM results. We adjusted the thickness of Al_2_O_3_ as shown in Figure 11 to meet the different thickness values of AAO60, AAO70, and AAO8 as shown in Figure 11a, Figure 11b, and Figure 11c, respectively. Transmittance increases with decreasing Al thickness in the three samples. However, the transmittance of cases with low Al thickness is still less than that of cases without Al, as shown in Figure 10. These results show that the plasmonic effect contribution does not exist.

## Conclusion

We produced AAO nanostructures on high-efficiency FC-BLEDs and found that various pore-widening durations can enhance LEE by approximately 1.6–2.9%. Material analysis revealed that AAO structures are destroyed after the second pore widening process. Thus, LEE is not enhanced by insufficient or excessive pore-widening durations. The mechanism underlying the blue shift of the emission wavelength in the AAO structures of FC-BLEDs remains unclear. The AAO process enhances the LEE of LEDs by reducing the total light reflected by the surface roughness of LEDs. RCWA analysis indicated that surface plasmon waves are not involved in LEE enhancement.

## Experimental

To produce a typical LED chip with a single-sided PSS, InGaN/GaN MQW LEDs were grown on c-plane (0001)-oriented PSSs using a metal–organic chemical vapor deposition apparatus. An inductively coupled plasma etcher was used to prepare periodic arrays with a depth of 1.5 μm on the PSS. Trimethylgallium, trimethylindium, ammonia, bicyclopentadienyl magnesium, and silane served as the precursors of Ga, In, N, Mg, and Si, respectively. The epitaxial structure of the LEDs was grown in accordance with a previously published procedure [8]. The textured surface of the PSS occurred at the initial growth site of a GaN nucleation layer. A thick n-type GaN layer (4 μm) doped with Si, a ten-period InGaN/GaN heterostructure used as the MQW, and a p-type GaN layer (120 nm) doped with Mg were vertically arranged on the nucleation layer in sequence. In the AAO process, a plate layer of aluminum film with a thickness of 2 μm was coated onto the LED surface, and a 0.3 M oxalic acid solution was prepared. The LED was placed into the solution to act as the anode, and a platinum sheet was used as the cathode.

**Figure 12:**
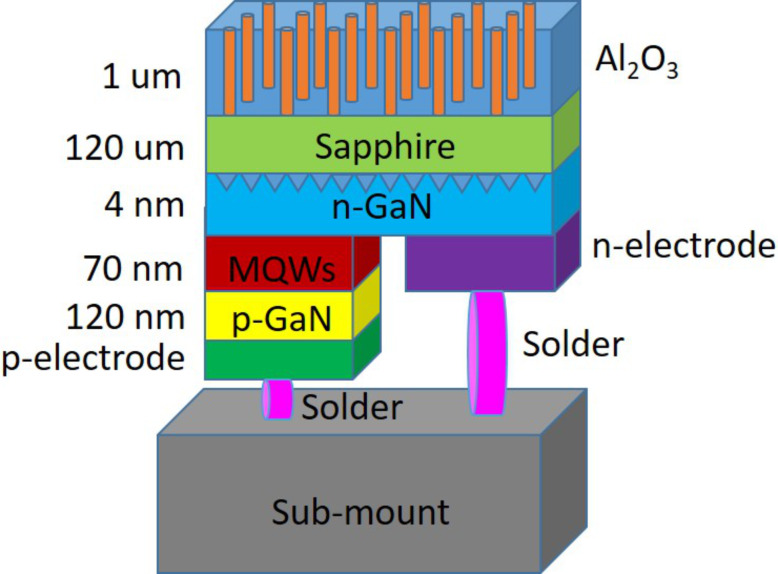
Structure of flip-chip LED with anodic aluminum oxide (AAO) processing.

The electrolysis experiment was conducted under 40 V with an electrolysis time of 45 min. Then, the system was placed in a mixed solution of 6 wt % phosphoric acid and 1.5 wt % chromic acid, and the first pore-widening process (1 h) and the second electrolysis process (45 min) were carried out. The second pore-widening process was conducted to eliminate possible residues in the barrier layer (Al_2_O_3_) of the AAO [47–49]. The second pore widening process was performed with durations of 60, 70, or 80 min, and the resulting samples were denoted as AAO60, AAO70, and AAO80, respectively. The device structure is shown in Figure 12 after the AAO processes are completed.
